# Molecular Markers for Granulovacuolar Degeneration Are Present in Rimmed Vacuoles

**DOI:** 10.1371/journal.pone.0080995

**Published:** 2013-11-28

**Authors:** Masahiro Nakamori, Tetsuya Takahashi, Tomokazu Nishikawa, Yu Yamazaki, Takashi Kurashige, Hirofumi Maruyama, Koji Arihiro, Masayasu Matsumoto

**Affiliations:** 1 Department of Clinical Neuroscience and Therapeutics, Hiroshima University Graduate School of Biomedical and Health Sciences, Hiroshima, Japan; 2 Department of Anatomical Pathology, Hiroshima University Graduate School of Biomedical and Health Sciences, Hiroshima, Japan; Brigham and Women's Hospital, Harvard Medical School, United States of America

## Abstract

**Background:**

Rimmed vacuoles (RVs) are round-oval cytoplasmic inclusions, detected in muscle cells of patients with myopathies, such as inclusion body myositis (IBM) and distal myopathy with RVs (DMRV). Granulovacuolar degeneration (GVD) bodies are spherical vacuoles containing argentophilic and hematoxyphilic granules, and are one of the pathological hallmarks commonly found in hippocampal pyramidal neurons of patients with aging-related neurodegenerative diseases, such as Alzheimer's disease and Parkinson's disease. These diseases are common in the elderly and share some pathological features. Therefore, we hypothesized that mechanisms of vacuolar formation in RVs and GVD bodies are common despite their role in two differing pathologies. We explored the components of RVs by immunohistochemistry, using antibodies for GVD markers.

**Methods:**

Subjects included one AD case, eight cases of sporadic IBM, and three cases of DMRV. We compared immunoreactivity and staining patterns for GVD markers. These markers included: (1) tau-modifying proteins (caspase 3, cyclin-dependent kinase 5 [CDK5], casein kinase 1δ [CK1δ], and c-jun N-terminal kinase [JNK]), (2) lipid raft-associated materials (annexin 2, leucine-rich repeat kinase 2 [LRRK2], and flotillin-1), and (3) other markers (charged multi-vesicular body protein 2B [CHMP2B] and phosphorylated transactive response DNA binding protein-43 [pTDP43]) in both GVD bodies and RVs. Furthermore, we performed double staining of each GVD marker with pTDP43 to verify the co-localization.

**Results:**

GVD markers, including lipid raft-associated proteins and tau kinases, were detected in RVs. CHMP2B, pTDP43, caspase 3, LRRK2, annexin 2 and flotillin-1 were detected on the rim and were diffusely distributed in the cytoplasm of RV-positive fibers. CDK5, CK1δ and JNK were detected only on the rim. In double staining experiments, all GVD markers colocalized with pTDP43 in RVs.

**Conclusions:**

These results suggest that RVs of muscle cells and GVD bodies of neurons share a number of molecules, such as raft-related proteins and tau-modifying proteins.

## Introduction

Rimmed vacuoles (RVs) are present in several myopathies, such as distal myopathy with RV formation (DMRV), inclusion-body myositis (IBM) [1], Becker muscular dystrophy [2], and oculopharyngeal dystrophy [3]. RVs consist of vacuoles surrounded by filamentous material forming round-oval or cleft-like shapes, and measure 3–20 µm in diameter. Most vacuoles are empty but some contain granules [1]. Sporadic IBM (s-IBM) is one of the most common muscle diseases, with prominent RVs in persons aged >50 years [4]. Furthermore, IBM muscle tissue shares phenotypic similarities with brain tissue of aging-related diseases, such as Alzheimer's disease (AD) and Parkinson's disease [4]. Vacuolar degeneration of muscle fibers in IBM is accompanied by multi-protein aggregates, such as β-amyloid (Aβ), phosphorylated tau (p-tau) in the form of paired helical filaments similar to degenerative hippocampal pyramidal cells in AD in regard to proteasome inhibition, endoplasmic reticulum stress, and lysosomal degradation [5], [6]. RVs consist of a number of proteins: cyclin-dependent kinase 5 (CDK5) [7], microtubule-associated protein (MAP) light chain3 (LC3) [8], histone H1 and other nuclear proteins [9], aquaporin-4 (AQP4) [10], O-linked N-acetylglucosamine [11], and optineurin. These proteins colocalize with phosphorylated transactive response DNA binding protein-43 (pTDP-43) in RVs, and the cytoplasm of RV-positive fibers [12]. RVs have been reported to be a by-product of an abnormally induced autophagic process [8], [13]–[15].

Granulovacuolar degeneration (GVD) bodies are one of the pathological hallmarks in hippocampal pyramidal neurons of AD [16], manifesting as small electron-dense inclusions of spherical vacuoles (3–5 µm diameter) containing argentophilic and hematoxyphilic granules [17]. However, the GVD body is not an AD-specific hallmark, but is observed during hippocampal p-tau accumulation in various neurodegenerative diseases, such as progressive supranuclear palsy, corticobasal degeneration, Pick's disease and pantothenate kinase-associated neurodegeneration, and in the normally aged brain [18]. Various proteins, such as casein kinase 1 (CK1) [19], glycogen-synthase kinase-3β (GSK3β) [20], c-jun N-terminal kinase (JNK) [21] and CDK5 [22] are thought to be involved in the pathophysiological mechanisms underlying the formation of GVD bodies by phosphorylating tau. Furthermore, activated caspase 3 [23], phospho-Smad2/3 [24], and pTDP43 [25], [26] are found in GVD bodies. Charged multivesicular body protein 2B (CHMP2B) is a subunit of the protein endosomal sorting complex required for transport (ESCRT)-III. CHMP2B shares a role in the transport of ubiquitinated protein to lysosomes in the autophagy–lysosomal pathway [27]. Lysosome-associated membrane protein 1 (LAMP1) is a late-stage autophagic marker [28], which also exists in GVD bodies. Therefore, GVD body formation is related to the autophagic pathway.

In addition to the accumulation of Aβ and tau in both hippocampal neurons and muscle cells, these autophagic vacuoles, RVs and GVD bodies show immunopositivity for both CDK5 [29] and pTDP43 [12], [30], [31]. These findings may suggest the existence of a common pathway in the formation of autophagic vacuole in the different organs and diseases [32]. However, studies testing this hypothesis have not been performed thus far. Therefore, in the current study, we explored the compositional similarities between RVs and GVD bodies by immunohistochemistry using antibodies for known GVD markers.

## Materials and Methods

### Ethics Statement

The protocols for neuropathological procedures and analyses were approved by and performed under the guidelines of the ethics committee of Hiroshima University, Graduate School of Biomedical and Health Sciences. Samples were obtained with the understanding and written informed consent of patients except for AD cases. AD samples were obtained with the understanding and written informed consent of family members. For this study, all samples were coded and personal information was dissociated from the test results. All data were analyzed anonymously, and all neuropathological procedures and analyses were conducted according to the principles expressed in the Declaration of Helsinki.

### Sample collection and tissue specimens

Hippocampal tissue specimens were obtained by autopsy from one AD case. Muscle tissue specimens were retrieved by biopsy from eight cases of s-IBM and three cases of DMRV formation (Table 1). In accordance with the National Institute on Aging-Alzheimer's Association guidelines for neuropathological assessment of AD, quantitative neuropathological criteria for diagnosis of the 73-year-old male with AD were fulfilled. For a high level of AD neuropathological change, these criteria indicated: (1) A3: an Aβ phase of 5, (2) B3: a neurofibrillary tangle (NFT) stage of 6, (3) C3: a score of ‘frequent’ in accordance with the Consortium to Establish a Registry for AD [33]. Within 24 hours of death, the hippocampus was extracted during autopsy, and fixed in 10% (v/v) formalin for 3 weeks. Paraffin-embedded sections (thickness 7 µm) were then prepared for subsequent procedures. Eight cases of s-IBM fulfilled the clinical and histopathological diagnostic criteria as well as no family history of IBM, as previously described [34]. In all three cases of DMRV, an UDP-N-acetylglucosamine 2-epimerase/N-acetylmannosamine kinase (GNE) gene mutation was confirmed by direct sequencing. Muscle specimens were extracted through biopsy, immediately frozen in isopentane cooled with liquid nitrogen, followed by the preparation of section (thickness 7 µm).

**Table 1:** Subject characteristics.

Case no.	Diagnosis	Sex	Age
sI-1	s-IBM	M	43
sI-2	s-IBM	M	57
sI-3	s-IBM	M	76
sI-4	s-IBM	M	51
sI-5	s-IBM	F	78
sI-6	s-IBM	F	80
sI-7	s-IBM	F	81
sI-8	s-IBM	M	69
D-1	DMRV	M	30
D-2	DMRV	F	37
D-3	DMRV	M	31

s-IBM, sporadic inclusion body myositis; DMRV, distal myopathy with rimmed vacuoles; F, female; M, male

### Antibodies

The antibodies used for immunohistochemistry and immunofluorescence studies are listed in Table 2. Two independent antibodies for the identification for each protein were used whenever possible to confirm the results from both staining techniques (anti-caspase 3, anti-CK1δ, anti-JNK, anti-annexin2, anti-flotillin-1 and anti-pTDP43 antibodies).

**Table 2:** List of primary antibodies.

Antigen	Clone	Subclass	Source	Animal	Dilution
CHMP2B	polyclonal	IgG	Abcam	rabbit	600
caspase3	polyclonal	IgG	Cell Signaling	rabbit	200
caspase3	polyclonal	IgG	Santa Cruz	rabbit	50
CDK5	polyclonal	IgG	Santa Cruz	rabbit	300
CK1δ	monoclonal	IgG	Santa Cruz	mouse	50
CK1δ	monoclonal	IgG	Abcam	mouse	500
JNK	polyclonal	IgG	Cell Signaling	rabbit	50
JNK	monoclonal	IgG	Santa Cruz	mouse	50
annexin2	polyclonal	IgG	Abcam	rabbit	200
annexin2	monoclonal	IgG	Santa Cruz	mouse	50
LRRK2	polyclonal	IgG	Novus Biologicals	rabbit	200
flotillin-1	monoclonal	IgG	BD bioscience	mouse	200
flotillin-1	polyclonal	IgG	Santa Cruz	rabbit	50
pTDP43	monoclonal	IgG	Cosmo Bio	mouse	3000
pTDP43	polyclonal	IgG	Cosmo Bio	rabbit	6000

### Immunohistochemistry using 3, 3-diaminobenzidine (DAB)

Hippocampal sections were deparaffinized and dehydrated. Muscle sections were subjected to antigen retrieval (microwave-heated in distilled water for 10 min) followed by washes in phosphate-buffered saline (PBS) for 3 min. Deparaffinized sections were then incubated in 3% H_2_O_2_/PBS for 60 min to eliminate endogenous peroxidase activity in tissues. Each section was incubated with primary antibodies overnight at 4°C. The sections were then washed three times in PBS and incubated with horseradish peroxidase (HRP)-conjugated anti-mouse or anti-rabbit antibodies for 30 min at room temperature. After sections were washed (three times in PBS), they were incubated at room temperature with DAB (Dako, Glostrup, Denmark).

### Immunofluorescence staining

Double staining on sections was performed, including on those of muscle containing RVs for further characterization. The same primary antibodies as listed in Table 1 were used in combination with rabbit or mouse antibody for pTDP43. For anti-CK1δ, anti-flotillin-1 and anti-pTDP43 primary antibodies, the following secondary antibodies were used: Alexa Fluor 488 donkey anti-mouse IgG or Alexa Fluor 546 donkey anti-rabbit IgG (all 1∶1000) (Molecular Probes, Eugene, OR, USA). The other primary antibodies with anti-pTDP43 were detected using the same HRP-conjugated secondary antibodies as described above, together with the TSA™KIT #12: Alexa Fluor 488 donkey, and TSA™ KIT #4: Alexa Fluor 568 donkey (Invitrogen, Eugene, OR, USA). Slides were mounted with Vectashield (Vector Laboratories, Burlingame, CA) and observed under the LSM510 confocal laser scanning microscope (Carl Zeiss AG, Oberkochen, Germany).

### Identification of RVs

Modified Gomori trichrome staining with anti-CHMP2B antibody was performed to verify RVs on serial sections prior to immunohistochemical staining.

## Results

### CHMP2B-positive vacuoles corresponded to RVs in muscles from patients with s-IBM and DMRV

**Figure 1:**
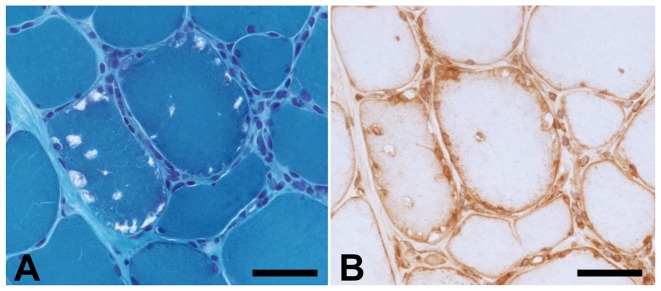
CHMP2B-positive structures corresponding to RVs. Modified Gomori trichrome staining, and immunohistochemical staining of serial sections using anti-charged multivesicular body protein 2B (CHMP2B) antibody, in an sporadic inclusion body myositis (s-IBM) case (case 1). Rimmed vacuoles (RVs) identified by modified Gomori trichrome staining (A) and the same structure immunopositive for CHMP2B (B). Scale bars  = 20 µm.

**Figure 2:**
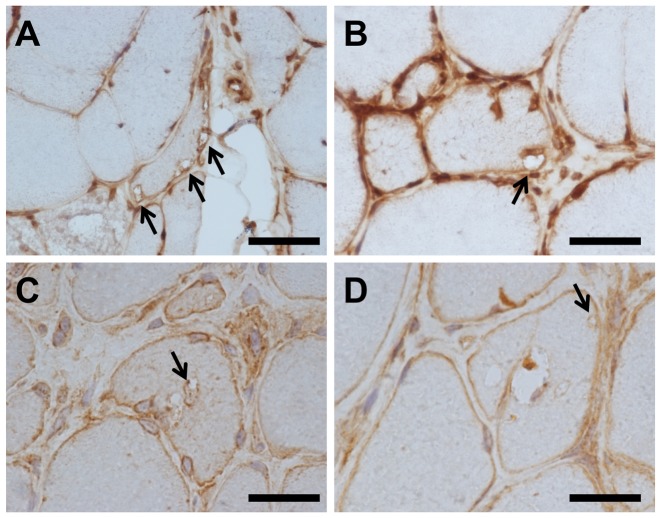
Immunohistochemistry for CHMP2B in muscle fibers of s-IBM and DMRV cases. RVs in all s-IBM (A–C) and distal myopathy with RVs (DMRV) (D) cases detected by anti- CHMP2B antibody. Arrows indicate RVs. Scale bar  = 20 µm.

Modified Gomori trichrome staining, and immunohistochemical staining of serial sections using anti-CHMP2B antibody, were performed. Gomori trichrome staining revealed RVs, which was confirmed by anti-CHMP2B (Fig. 1). The anti-CHMP2B antibody in DMRV and s-IBM cases revealed RVs and sarcolemma (Fig. 2).

### RVs were immunopositive for GVD markers

**Figure 3:**
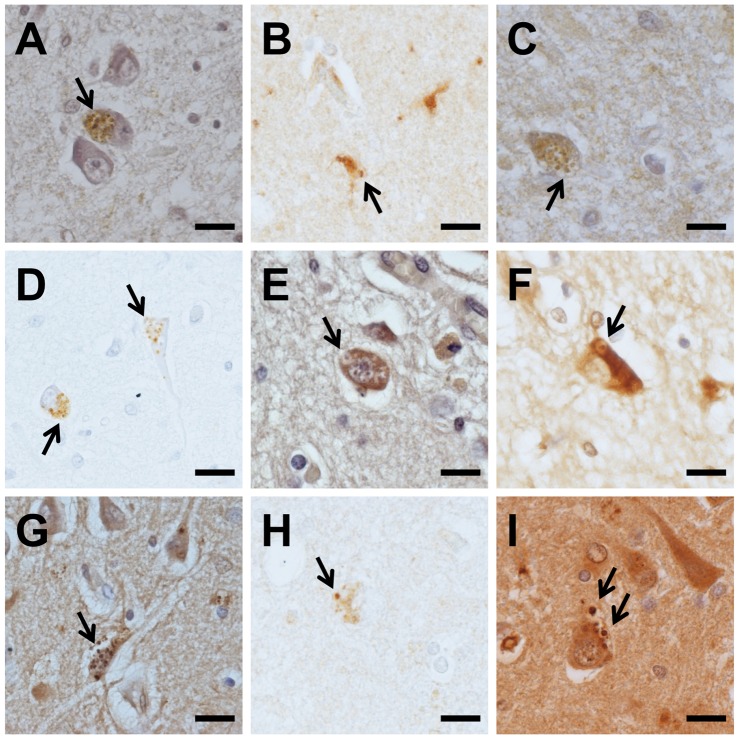
Immunohistochemistry for GVD markers in the Alzheimer's disease hippocampus. Anti-CHMP2B (A), anti-caspase3 (B), anti-cyclin-dependent kinase 5 (CDK5) (C), anti-casein kinase 1δ (CK1δ) (D), anti-c-jun N-terminal kinase (JNK) (E), anti-leucine-rich repeat kinase 2 (LRRK2) (F), anti-annexin2 (G), anti-flotillin-1 (H), and anti-phosphorylated transactive response DNA binding protein-43 (pTDP43) (I). Arrows indicate granulovacuolar degeneration (GVD) bodies. Scale bars  = 20 µm.

We next compared the immunoreactivity of RVs in the hippocampus of an AD case with those of muscle tissue, using antibodies against reported GVD markers (tau-modifying proteins, lipid raft-associated materials, CHMP2B and pTDP43). GVD bodies were immunopositive for all antibodies and mainly found in the CA1 subregion of the hippocampus (Fig. 3). In pyramidal cells, caspase 3, CDK5, JNK, annexin 2, LRRK2, flotillin-1 and pTDP43 were diffusely distributed in the cytoplasm, forming fine spherical granules, as well as GVD bodies (Fig. 3). NFTs were immunopositive for caspase 3, CDK5, LRRK2, annexin 2, flotillin-1 and pTDP43.

**Figure 4:**
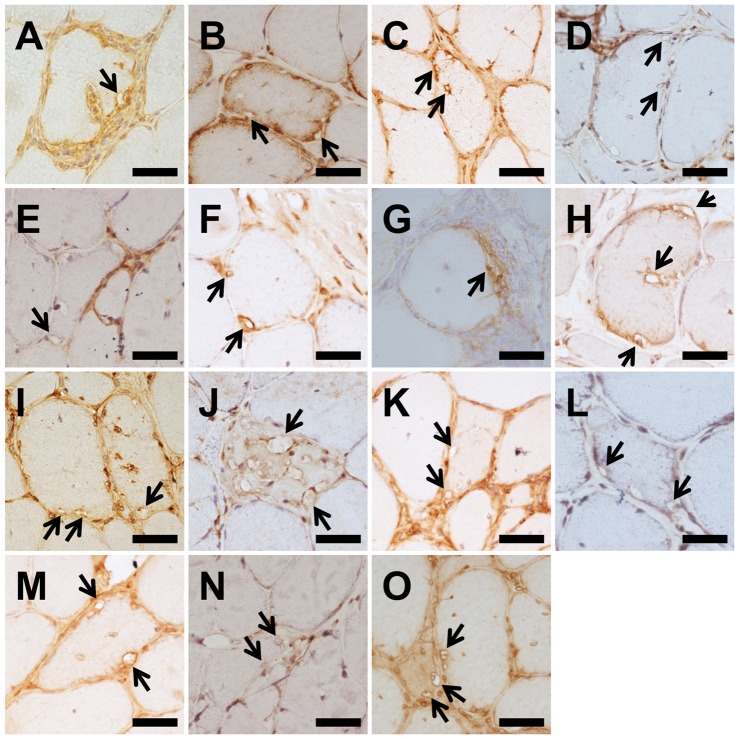
Immunohistochemistry for GVD markers in muscle fibers of s-IBM cases. Anti-CHMP2B (A), anti-caspase3 (B, C), anti-CDK5 (D), anti- CK1δ (E, F), anti-JNK (G, H), anti-LRRK2 (I), anti-annexin2 (J, K), anti-flotillin-1 (L, M), and anti-pTDP43 (N, O). (B, C), (E, F), (G,H), (J, K), (L, M) and (N, O) indicate 2 independent antibodies for the identification of each protein. Arrows indicate RVs. Scale bar  = 20 µm.

RVs were mainly found in degenerated, atrophic muscle fibers in all s-IBM and DMRV cases (Fig. 4). Some vacuoles contained granules resembling intraluminal vesicles of GVD, both of which were immunopositive for all of markers listed in Table 2. CHMP2B, caspase 3 and pTDP43 were diffusely distributed in the cytoplasm of RV-positive fiber as well as on the rim (Fig. 4). Moreover, CHMP2B and caspase 3 were detected in sarcolemma of most muscle fibers (Fig. 4). CDK5, CK1δ and JNK were only detected on the rim and scarcely present in other regions of RV-positive fibers (Fig. 4). LRRK2, annexin 2 and flotillin-1 were present on the rim and diffusely distributed in the cytoplasm of RV-positive fibers (Fig. 4).

Furthermore, the rims of some vacuole-containing nuclei were immunopositive for all of these markers, but the nuclei themselves were not immunostained. Infiltrating inflammatory cells were not stained (Fig. 4).

### All GVD markers colocalized with pTDP43 in RVs

**Figure 5:**
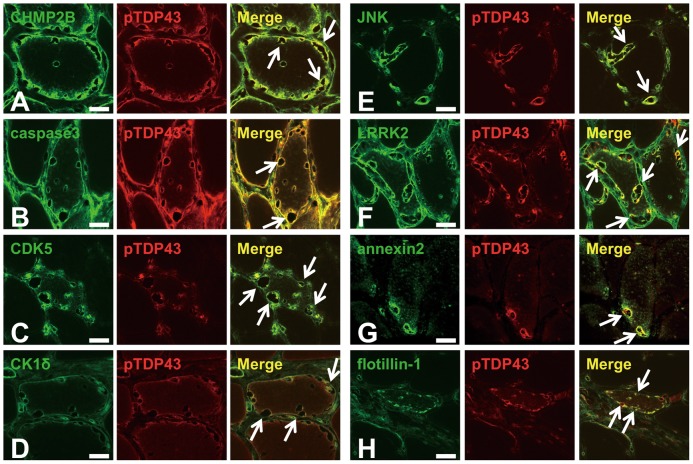
Representative confocal laser scanning micrograph. Double immunofluorescence labeling and merged images in muscle sections from patients with s-IBM for CHMP2B (green) and pTDP43 (red) (A), caspase3 (green) and pTDP43 (red) (B), CDK5 (green) and pTDP43 (red) (C), CK1δ (green) and pTDP43 (red) (D), JNK (green) and pTDP43 (red) (E), LRRK2 (green) and pTDP43 (red) (F), annexin 2 (green) and pTDP43 (red) (G), and flotillin-1 (green) and pTDP43 (red) (H). Arrows indicate RVs. Scale bar  = 20 µm.

Because pTDP43 is present in RVs [12], [30], [31] and GVD bodies [35], we therefore performed double immunofluorescence staining using anti-pTDP43 antibody together with antibodies for CHMP2B, caspase 3, CDK5, CK1δ, JNK, LRRK2, annexin 2 and flotillin-1 in s-IBM cases. Results showed that pTDP43 colocalized with all of these markers in RVs (Fig. 5). These results suggested that numerous GVD markers were present in RVs.

## Discussion

The present study reveals a number of GVD markers present in RVs, namely raft-related proteins and tau-modifying proteins. These findings suggest that RVs are similar in nature to GVD bodies, and that GVD bodies and RVs partly exhibit a common origin.

The lipid raft is a subdomain of the plasma membrane containing high concentrations of cholesterol and glycosphingolipids, thus providing a platform for cell signaling [36]. Phosphatidylinositol 4,5-bisphosphate (PtdIns[4], [5]P2) is another lipid component of the lipid raft [37], and colocalizes with proteins, such as flotillin-1 and annexin 2 (both lipid raft markers) [38]–[40]. Recently, lipid rafts have been reported to play a role in endocytic pathways independent of the clathrin pathway [41]. Furthermore, lipid raft vacuoles have been shown to be transported to the autophagosome via the ESCRT pathway [42], [43]. The raft-dependent pathway is also involved in the formation of intracellular Aβ deposits [44]–[46]. Several lines of evidence show that intracellular Aβ is significantly more toxic than extracellular Aβ in disrupting synaptic activity and facilitating the hyperphosphorylation of tau in the AD brain [46]. We have previously reported that GVD bodies are immunopositive for PtdIns(4,5)P2, flotillin-1, annexin2 and LRRK2 [40], indicating that GVD bodies are autophagic vacuoles with a raft component. In the current study, we demonstrated that these raft markers were also evident in RVs. In addition to the presence of raft-related proteins in GVD bodies, immunoreactivity of RVs for raft markers suggest that RVs are raft-dependent structures like GVD bodies.

MAP tau is phosphorylated by a number of kinases, such as CK1 [19], GSK3β [20], JNK [21], and cleaved by caspase 3 [47]. These tau-modifying proteins are present in GVD bodies. In contrast, CDK5, another tau kinase, is present in RVs [9]. Recently, we have reported that CDK5 is a novel marker for GVD bodies [22]. In the present study, a number of tau-modifying proteins (i.e. GVD markers) were present in RVs. In addition to the presence of CDK5 in GVD bodies, immunoreactivity of RVs for other tau-modifying proteins suggests RVs are structures related to tau modification like GVD bodies.

We and others have previously shown that CHMP2B is present in GVD bodies [27], [28], thus suggesting that GVD bodies may be generated as multivesicular bodies in the endocytic pathway. Morphological observations via electron microscopy suggest that GVD bodies are an age-related and unique form of autophagic vacuole, and that intraluminal vesicles are residues of the autophagic process [17]. RVs are also reported to be derived from autophagic vacuoles [13]. For example, use of LC3 antibodies indicated that RVs were involved in autophagosome formation [8]. Because both GVD bodies and RVs are vacuoles likely to be derived from autophagic mechanisms, they may thus share similar structural components. In the current study, we revealed that markers of GVD bodies: CHMP2B, lipid raft-associated proteins and tau-modifying proteins, were present in RVs. Some of these markers were only localized in RVs, and others were diffusely distributed in the cytoplasm or sarcolemma as well as in RVs. Although the immunoreactivity of GVD markers for RVs and GVD bodies in this study did not differ significantly, differences between RVs and GVD bodies have been previously reported. RV formation may be related to early-stage autophagic organelles as shown in their immunoreactivity for LC3 at this time-point [8]. Conversely, GVD bodies may be predominantly related to late-stage autophagic organelles, evidenced by their immunoreactivity for LAMP1 and cathepsin D, and weak signals for early-stage autophagic markers [28]. Further study is required to determine the materials associated with the autophagy and endosome pathways, as well as the tau phosphorylation pathway, to ascertain the differences in components between RVs and GVD bodies.

Immunohistochemistry for selected proteins may have been a limitation of the present study. An attempt at a comprehensive analysis on the composition of both structures using imaging mass spectrometry was unsuccessful because of limited resolution. Further advances in technology would therefore enable us to perform extensive analyses of RVs and GVD bodies in the future.

## Conclusions

Our results suggest that RVs of muscle cells and GVD bodies of neurons have a number of common molecules, such as raft-related proteins and tau-modifying proteins. Further investigations will elucidate more precisely the mechanisms by which vacuolar pathological structures are generated, including aging-related changes.
